# Young infant sepsis: aetiology, antibiotic susceptibility and clinical signs

**DOI:** 10.1016/j.trstmh.2007.05.005

**Published:** 2007-10

**Authors:** Opiyo Newton, Mike English

**Affiliations:** a Kenya Medical Research Institute/Wellcome Trust Research Programme, P.O. Box 43640, 00100 GPO, Nairobi, Kenya; b Department of Paediatrics, University of Oxford, Oxford, UK

**Keywords:** Sepsis, Aetiology, Antibiotics, Signs, Bacterial infection, Infant

## Abstract

Globally, young infant mortality comprises 40% of the estimated 10.8 million child deaths annually. Almost all (99%) of these deaths arise in low- and middle-income countries (LMICs). Achievement of the Millennium Development Goal for child survival, however, requires a significant improvement in the management of infections in young infants. We have reviewed current evidence from LMICs on one major cause of young infant mortality, severe infection, and have described the range of pathogens, reported antibiotic susceptibility and value of clinical signs in identifying severe bacterial illness. Evidence from the reviewed studies appears to show that common pathogens in young infant infections change over time and vary within and across settings. However, there are few good, large studies outside major urban settings and many reports describe infections of babies born in hospital when most young infant infections probably occur in the majority born at home. Yet what knowledge there is can aid in instituting prompt and appropriate therapy, and perhaps thus minimize the emergence of multidrug-resistant bacteraemia, a major threat at least in hospital settings. Improved country level data on pattern of microorganisms, resistance and antibiotic use are required to help reduce mortality through development of local, evidence-based clinical guidelines.

## 1 Introduction

Despite overall improvement in the health of children worldwide, mortality among young infants less than 2 months old remains high (Bhutta et al., 2003). Young infant mortality comprises 40% of the estimated 10.8 million child deaths worldwide annually (Duke et al., 2005). About 4 million of these deaths (more than 10 000 deaths per day) occur during the neonatal period, with severe infections being a direct cause in 26% of them (Lawn et al., 2005). Nearly all of these deaths (99%) arise in low- and middle-income countries (LMICs). Thus, in LMICs, the fourth Millennium Development Goal, which aspires to a global target, by 2015, of reducing the under five mortality by two-thirds, cannot be achieved without substantial reductions in neonatal deaths (Lawn et al., 2005, Zupan, 2005).

Addressing young infant mortality attributable to infection calls for a continuum of care, including, importantly, better prevention, improved recognition of severe illness and appropriate case management. The latter should take account of the site at which the infections are acquired (hospital or community) (Zaidi et al., 2005) and their time of onset (early or late) (Vergnano et al., 2005). Both these factors may have implications for the choice of empiric antibiotic therapy. In addition, quick identification and referral of the severely sick infants from the home or primary care setting may be life-saving. We therefore summarized current evidence on the aetiology, antibiotic susceptibility and clinical signs in young infant sepsis that might support the introduction of evidence-based practice guidelines.

## 2 Materials and methods

### 2.1 Clinical questions

We conducted literature searches to answer clinical questions on the aetiology, antibiotic susceptibility and clinical signs in young infant sepsis (Table 1). We aimed to include only recent data from the LMICs.

**Table 1:** Clinical questions

1. What are the major bacterial pathogens in the neonatal/young infant age group?
2. What is known about antibiotic sensitivities/resistance in common pathogens in young infants?
3. What clinical signs best identify severe illness in young infants?

### 2.2 Search strategy

Potential articles for inclusion were identified by direct searches of MEDLINE via PubMed by use of clinical queries (1990 to March 2006) (Haynes and Wilcynski, 2004, Montori et al., 2005) and without language restriction. We used the following combinations of search terms:

• Aetiology, broad sensitive search: *neonatal sepsis AND (bacterial pathogens OR bacterial isolates)*
• Therapy, broad sensitive search: *neonatal sepsis AND (bacterial susceptibility OR antibiotic sensitivity OR antibiotic resistance)*
• Diagnosis, narrow specific search: *(sepsis OR severe illness) AND (signs OR predictors) AND (neonates OR infants)*

**Figure 1:**
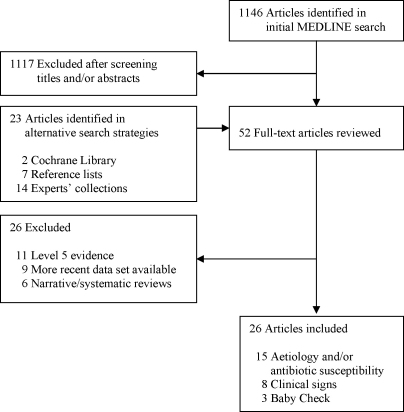
Study identification and selection process.

To ensure a more comprehensive review, we conducted supplementary searches in the Cochrane Library (Issue 2, 2006), bibliographies of retrieved articles and reference collection of experts in the field (Figure 1).

### 2.3 Study selection and quality assessment

The titles and abstracts of the retrieved articles were read by two independent reviewers. Studies from LMICs meeting the following inclusion criteria were selected for a more detailed review: those with primary data on aetiological agents, antibiotic sensitivity/resistance and clinical signs predicting severe illness in infants less than 2 months. For studies of clinical signs, the search was expanded to include those from all countries. Existing high quality reviews were not replicated, but individual studies included in these reviews were re-examined, if required, to answer our particular clinical questions. Articles were not excluded if the studies enrolled some children outside the 0–2 months age range. However, articles on biochemical markers of severe illness were excluded, as such laboratory investigations are rarely available in LMICs.

The methodological quality of the studies was assessed using the Oxford Centre for Evidence-Based Medicine levels of evidence, which ranks the validity of evidence in a hierarchy of levels, with systematic reviews (SRs) as level 1 (strong evidence) and expert opinions as level 5 (weak evidence) (Phillips et al., 1988).

## 3 Results

### 3.1 Description of included studies

Overall, we identified 25 articles that met the preset inclusion criteria: 14 of the articles were on aetiology (Adejuyigbe et al., 2004, Aurangzeb and Hameed, 2003, Bell et al., 2005, Berkley et al., 2005, Chacko and Sohi, 2005, Jiang et al., 2004, Kaushik et al., 1998, Laving et al., 2003, Milledge et al., 2005, Mokuolu et al., 2002, Musoke and Revathi, 2000, Rahman et al., 2002, Waheed et al., 2003, WHO Young Infants Study Group, 1999); 10 had data on antibiotic sensitivities/resistances (Adejuyigbe et al., 2004, Aurangzeb and Hameed, 2003, Berkley et al., 2005, Duke et al., 2005, Laving et al., 2003, Milledge et al., 2005, Mokuolu et al., 2002, Musoke and Revathi, 2000, Rahman et al., 2002, WHO Young Infants Study Group, 1999); eight assessed clinical signs of serious infections in young infants (Bang et al., 2005, Duke et al., 2005, English et al., 2003, English et al., 2004, Gupta et al., 2000, Hewson et al., 2000, Kalter et al., 1997, Weber et al., 2003); and three examined the Baby Check scoring system (Chandran et al., 1998, Morley et al., 1994, Twite and Morley, 1995). Ten articles (Adejuyigbe et al., 2004, Aurangzeb and Hameed, 2003, Berkley et al., 2005, Duke et al., 2005, Laving et al., 2003, Milledge et al., 2005, Mokuolu et al., 2002, Musoke and Revathi, 2000, Rahman et al., 2002, WHO Young Infants Study Group, 1999) examined more than one aspect of the clinical questions and are therefore included more than once in this summary. Seventeen studies were prospective (Adejuyigbe et al., 2004, Aurangzeb and Hameed, 2003, Berkley et al., 2005, Chacko and Sohi, 2005, Chandran et al., 1998, Duke et al., 2005, English et al., 2003, English et al., 2004, Gupta et al., 2000, Hewson et al., 2000, Kalter et al., 1997, Weber et al., 2003, Kaushik et al., 1998, Morley et al., 1994, Rahman et al., 2002, Twite and Morley, 1995, WHO Young Infants Study Group, 1999); six were retrospective (Bang et al., 2005, Bell et al., 2005, Clark et al., 2006, Jiang et al., 2004, Milledge et al., 2005, Mokuolu et al., 2002); two were descriptive (Laving et al., 2003, Waheed et al., 2003); and one was partly prospective and partly retrospective (Musoke and Revathi, 2000). Four studies included some infants outside the 0–2 months age group (Berkley et al., 2005, English et al., 2003, Hewson et al., 2000, WHO Young Infants Study Group, 1999). The findings of the studies on aetiology and antibiotic sensitivities/resistances were categorized by setting (hospital-acquired, community-acquired, mixed site) and time of onset (early onset, late onset).

### 3.2 Definitions

Severe illness was defined as one of: a positive blood culture or cerebrospinal fluid culture; a chest X-ray with consolidation or an oxygen saturation of <90% (English et al., 2003, English et al., 2004, Hewson et al., 2000, Weber et al., 2003, WHO Young Infants Study Group, 1999); death (English et al., 2004, Weber et al., 2003); or need for ‘significant inpatient treatment’ (fluid therapy, parental antibiotics, oxygen, surgery) (Hewson et al., 2000). Hospital-acquired infection was defined as any infection associated with birth or a stay in a hospital, while community- acquired infection was defined as any infection associated with probable home birth. Data described as from mixed sites are from studies where it is either not possible to tell whether pathogens reported to be responsible for young infant sepsis are hospital- or community-acquired or the report covers a truly mixed population with no stratification by likely origin of infection. Relative consistency in the applied definition is imperative for the comparison of data in the included studies.

### 3.3 Bacterial aetiology (Table 2)

Fourteen articles were reviewed to determine the spectrum of bacterial pathogens responsible for young infant infections and to compare these pathogens across settings. In one hospital-based study, *Escherichia coli* (78%; 52/67) was the major pathogen (Aurangzeb and Hameed, 2003). Data on community-acquired sepsis were scarce, however, with only two studies reported (Berkley et al., 2005, WHO Young Infants Study Group, 1999). The spectrum of organisms reported in these studies differ, with *E. coli* and Group B *Streptococcus* (GBS) dominating in one Kenyan study (Berkley et al., 2005) and *Streptococcus pneumoniae*, *Staphylococcus aureus*, *E. coli* and *Salmonella* predominating in the other (WHO Young Infants Study Group, 1999).

**Table 2:** Bacterial pathogens

Bacterial isolate		1	2	3	4	5	6	7	8	9	10	11	12	13	14	Total
Gram positive	*Staph. aureus*	87 (11.1)	11 (7.8)	16 (9.3)	2 (13.3)	153 (61.2)	44 (12.5)	–	–	23 (9.9)	18 (29.5)	9 (7.4)	296 (29.5)	29 (17.8)	–	688 (20.1)
	CONS^a^	–	13 (9.2)	–	–	47 (18.8)	71 (20.1)	–	–	–	15 (24.6)	–	–	–	–	146 (4.3)
	*Staph. epidermidis*	–	–	–	–	–	–	–	–	21 (9)	–	19 (15.7)	–	–	4 (10.0)	44 (1.3)
	Group B streptococci	136 (17.4)	15 (10.6)	20 (11.6)	–	–	18 (5.1)	–	4 (26.7)	–	–	–	–	3 (1.8)	–	196 (5.7)
	Group A streptococci	56 (7.1)	–	13 (7.5)	–	–	–	–	–	–	–	–	–	–	–	69 (2.0)
	Other streptococci	–	13 (9.2)	–	1 (6.7)	–	–	–	–	–	–	–	–	–	–	14 (0.4)
	*S. pneumoniae*	79 (10.1)	–	13 (7.5)	–	–	–	–	–	–	–	–	–	37 (22.7)	–	129 (3.8)
	Other	72 (9.2)	–	8 (4.6)	–	4 (1.6)	27 (7.5)	4 (5.9)	–	2 (0.86)	2 (3.3)	4 (3.3)	–	28 (17.2)	16 (40.0)	167 (4.9)

Gram negative	*Escherichia coli*	67 (8.6)	22 (15.6)	19 (11.0)	1 (6.7)	5 (2.0)	44 (12.5)	52 (77.6)	7 (46.7)	111 (47.5)	3 (4.9)	4 (3.3)	367 (36.6)	23 (14.1)	11 (27.5)	736 (21.5)
	*Pseudomonas*	–	4 (2.8)	9 (5.2)	9 (60.0)	6 (2.4)	20 (5.7)	6 (8.9)	–	37 (15.9)	1 (1.6)	–	225 (22.4)	4 (2.5)	–	321 (9.4)
	*Klebsiella*	60 (7.7)	39 (27.7)	17 (9.8)	2 (13.3)	2 (0.8)	25 (7.1)	5 (7.5)	2 (13.3)	39 (16.8)	10 (16.4)	38 (31.4)	77 (7.6)	3 (1.8)	6 (15.0)	325 (9.5)
	*Salmonella*	110 (14.0)	–	3 (1.7)	–	15 (6.0)	–	–	–	–	–	2 (1.7)	–	14 (8.6)	–	144 (4.2)
	*Enterobacter*	–	14 (9.9)	–	–	2 (0.8)	–	–	–	–	–	19 (15.7)	–	10 (6.1)	3 (7.5)	48 (1.4)
	*Acinetobacter*	–	2 (1.4)	17 (9.8)	–	–	26 (7.4)	–	–	–	3 (4.9)	–	–	3 (1.8)	–	51 (1.5)
	*Proteus*	–	3 (2.1)	–	–	13 (5.2)	–	–	–	–	–	–	38 (3.8)	–	–	54 (1.6)
	*Citrobacter*	–	2 (1.4)	–	–	3 (1.2)	–	–	–	–	–	–	–	–	–	5 (0.1)
	Other	117 (14.9)	3 (2.1)	38 (22.0)	–	–	61 (17.3)	–	–	–	9 (14.8)	26 (21.5)	–	5 (3.1)	–	259 (7.6)

Other pathogens	–	–	–	–	–	17 (4.8)	–	2 (13.3)	–	–	–	–	4 (2.5)	–	23 (0.7)	

Total		784	141	173	15	250	353	67	15	233	61	121	1003	163	40	3419

1: Milledge et al. (2005); 2: Bell et al. (2005); 3: Berkley et al. (2005); 4: Chacko and Sohi (2005); 5: Adejuyigbe et al. (2004); 6: Jiang et al. (2004); 7: Aurangzeb and Hameed (2003); 8: Laving et al. (2003); 9: Waheed et al. (2003); 10: Mokuolu et al. (2002); 11: Musoke et al. (2002); 12: Rahman et al. (2002); 13: WHO Young Infants Study Group (1999); 14: Kaushik et al. (1998). Data are no. (%) of culture-confirmed isolates. All the studies were of level 4 evidence.

a Coagulase-negative *Staphylococcus*.

Eight studies present mixed-site data on young infant infections (Adejuyigbe et al., 2004, Kaushik et al., 1998, Laving et al., 2003, Milledge et al., 2005, Mokuolu et al., 2002, Musoke and Revathi, 2000, Rahman et al., 2002, Waheed et al., 2003). Summarizing these data, gram-negative organisms were isolated more frequently than gram-positive organisms, with *E. coli* (Kaushik et al., 1998, Rahman et al., 2002), *Klebsiella* (Kaushik et al., 1998, Laving et al., 2003, Mokuolu et al., 2002, Musoke and Revathi, 2000), GBS (Laving et al., 2003, Milledge et al., 2005) and *Staph. aureus* (Adejuyigbe et al., 2004, Kaushik et al., 1998, Milledge et al., 2005, Mokuolu et al., 2002, Waheed et al., 2003) being the most frequently reported isolates.

#### 3.3.1 Bacterial pathogens causing early- and late-onset sepsis

Early-onset sepsis (EOS) is mainly acquired before delivery (vertical transmission from organisms that infect/colonize the maternal genital tract or via the placenta after maternal bacteraemia) or during delivery as the baby passes through the birth canal. By contrast, late-onset sepsis (LOS) is mostly acquired after delivery from hospital or community sources. This classification is thought to be helpful because of probable differences in the pathophysiology and pathogens associated with EOS and LOS. The clinical relevance of data stratified by time of onset of sepsis is, however, limited by wide variations in the age limits in the definitions [for EOS, 0–72 h (Chacko and Sohi, 2005, Kaushik et al., 1998) or 0–7 d (Aurangzeb and Hameed, 2003, Bell et al., 2005, Jiang et al., 2004, Waheed et al., 2003); for LOS, >72 h (Chacko and Sohi, 2005, Kaushik et al., 1998) or >7 d (Aurangzeb and Hameed, 2003, Bell et al., 2005, Jiang et al., 2004, Waheed et al., 2003)]. It was not possible to standardize the above definitions, so the original study definitions are retained.

#### 3.3.2 Hospital-acquired EOS and LOS

Two studies (Aurangzeb and Hameed, 2003, Chacko and Sohi, 2005) presented data on hospital-acquired EOS and LOS. Generally, gram-negative organisms were isolated significantly more frequently than gram-positives, with *Klebsiella*, *Pseudomonas*, *E. coli* and *Staph. aureus* predominating in both EOS and LOS. However, the similarity in the spectrum of organisms causing EOS and LOS is confounded by differences in the age limits in the studies included in the review.

#### 3.3.3 Mixed-site EOS and LOS

Data from five studies (Bell et al., 2005, Jiang et al., 2004, Kaushik et al., 1998, Mokuolu et al., 2002, Waheed et al., 2003) on mixed-site EOS and LOS show that *E. coli* and GBS are the common pathogens in EOS. However, the pathogens involved in LOS are varied and include *E. coli* (Bell et al., 2005, Jiang et al., 2004, Waheed et al., 2003), *Staph. aureus* (Aurangzeb and Hameed, 2003, Mokuolu et al., 2002) and coagulase-negative *Staphylococcus* (*CONS*) (Jiang et al., 2004, Mokuolu et al., 2002).

### 3.4 Antimicrobial susceptibility (Table 3)

In many developing countries, antimicrobial sensitivity and resistance testing, a necessary component of rational antimicrobial prescribing, is uncommon and may be unreliable in routine clinical settings. The gold standard for assessing antimicrobial susceptibility is determination of the minimum inhibitory concentration (MIC). Classically, this is undertaken using a labour-intensive serial dilution technique, although more recently an accurate quantitative and rapid technique (E-test) has been successfully used (Berkley et al., 2005). Among the studies reviewed, most report resistance rates based on simple disc diffusion, a technique that provides an ‘all-or-none’ classification that will not, especially in the case of penicillin class drugs, provide any indication of whether resistance is of an intermediate or high grade, which can be clinically very important. We therefore summarize the results of studies on antibiotic treatment regimens and susceptibilities, including the susceptibility testing method (Table 2), by settings.

**Table 3:** Antibiotic sensitivity

Pathogen	Antibiotic	1	2	3	4	5	6	Total
*Escherichia coli*	Ampicillin	17/67 (25)	2/5 (40)	10/52 (19.1)	–	0/2 (0)	40/367 (11)	69/528 (13.1)
	Amoxycillin	–	3/5 (60)	13/52 (25)	–	–	–	16/57 (28.1)
	Gentamicin	62/67 (93)	4/5 (80)	32/52 (61.5)	–	3/3 (100)	78/367 (21.3)	179/494 (36.2)
	Cefotaxime	–	–	20/52 (38.4)	72/111 (65)	–	120/367 (32.6)	212/530 (40.0)
	Ceftazidime	–	–	12/52 (23)	61/111 (55)	0/1 (0)	119/367 (32.5)	192/531 (36.2)
	Ceftriaxone	8/8 (100)	–	26/52 (50)	–	2/2 (100)	103/367 (28)	139/429 (32.4)

*Pseudomonas*	Ampicillin	–	3/5 (60)	2/6 (33.3)	–	–	31/225 (13.6)	36/236 (15.3)
	Amoxycillin	–	2/55 (40)	2/6 (33.3)	–	–	–	4/61 (6.6)
	Gentamicin	–	5/5 (100)	52/156 (33.3)	–	–	48/225 (21.4)	105/386 (27.2)
	Cefotaxime	–	–	4/6 (66.6)	19/37 (50)	–	61/225 (27)	84/268 (31.3)
	Ceftazidime	–	–	1/6 (16.6)	13/37 (35)	–	98/225 (43.5)	112/268 (41.8)
	Ceftriaxone	–	–	4/6 (66.6)	–	–	74/225 (33)	78/231 (33.8)

*Klebsiella*	Ampicillin	0/60 (0)	–	1/5 (20)	–	1/4 (25)	27/77 (34.7)	29/146 (19.9)
	Amoxycillin	–	0/1 (0)	0/5 (0)	–	–	–	0/6 (0)
	Gentamicin	20/60 (33)	1/1 (100)	2/5 (40)	–	6/9 (66.7)	12/77 (16)	41/152 (27.0)
	Cefotaxime	–	–	3/5 (60)	31/39 (80)	–	11/77 (14)	45/121 (37.2)
	Ceftazidime	–	–	3/5 (60)	31/39 (80)	2/4 (50)	26/77 (33.8)	62/125 (49.6)
	Ceftriaxone	4/4 (100)	–	3/5 (60)	–	5/6 (83.3)	14/77 (18)	26/92 (28.3)

*Staph. aureus*	Ampicillin	74/86 (86)	–	1/4 (25)	–	1/6 (16.7)	178/296 (60)	254/392 (64.8)
	Amoxycillin	–	74/101 (73)	2/4 (50)	–	–	–	76/105 (72.4)
	Gentamicin	–	87/101 (85.8)	2/4 (50)	–	7/12 (58.3)	89/296 (30)	185/413 (44.8)
	Cefotaxime	–	–	3/4 (75)	16/23 (70)	–	148/296 (50)	167/323 (51.8)
	Ceftazidime	–	–	3/4 (75)	16/23 (70)	5/7 (71.4)	109/296 (36.8)	133/330 (40.3)
	Ceftriaxone	–	–	3/4 (75)	–	6/11 (54.5)	122/296 (41.2)	131/311 (42.1)

1: Milledge et al. (2005); 2: Adejuyigbe et al. (2004); 3: Aurangzeb and Hameed (2003); 4: Waheed et al. (2003); 5: Mokuolu et al. (2002); 6: Rahman et al. (2002). Data are no. of isolates sensitive/no. tested (% sensitive).

#### 3.4.1 Community-based studies

In one Kenyan study on community-acquired invasive bacterial disease, in-vitro antimicrobial susceptibilities among all the bacteria were as follows: amoxycillin/ampicillin (55%); benzylpenicillin alone (31%); either penicillin or gentamicin (88%); either ampicillin or gentamicin (97%); and either penicillin or chloramphenicol (82%) (Berkley et al., 2005). These results support the findings of the multicentre WHO study on serious infections in young infants, which suggest initial therapy of neonatal sepsis with ampicillin and gentamicin (WHO Young Infants Study Group, 1999). This regimen, however, provides only modest cover for *Staph. aureus*. Thus, in suspected staphylococcal (skin) infection, initial therapy should include cloxacillin, but if this is used to replace ampicillin, the reduced efficacy of the combination against gram-negative infections should be kept in mind and failure to improve within 48 h should perhaps prompt use of the combination of cloxacillin, ampicillin and gentamicin.

#### 3.4.2 Mixed studies

Resistance of gram-negative organisms to the empiric first-line antibiotics remains high: ampicillin (79.3%), amoxicillin (74.6%) and gentamicin (43.2%) (Aurangzeb and Hameed, 2003). However, this finding requires validation, given that most of these data were collected from specialized newborn units, where levels of in-vitro resistance to antibiotics may be considerably higher than in smaller hospitals or for community-acquired infection. Gentamicin resistance among gram-negative bacteria is variable: 20% in Kenya; 24% in India; 43% in one study from Pakistan and 78–84% in another; 66% in Papua New Guinea; and 77% in Guatemala (Duke et al., 2005). These findings are consistent with those of another review, which reported that resistance rates of *E. coli*, *Klebsiella* and *Pseudomonas* ranged from 65 to 100% for ampicillin and 0 to 93% for gentamicin (Vergnano et al., 2005).

Additional data from Malawi (Milledge et al., 2005), Kenya (Laving et al., 2003) and Pakistan (Rahman et al., 2002) show that resistance rates of gram-negative organisms to ampicillin and gentamicin remain high. By contrast, in studies from Nigeria (Adejuyigbe et al., 2004, Mokuolu et al., 2002) and Kenya (Musoke and Revathi, 2000), gram-negative organisms showed good sensitivities to gentamicin and to amikacin, cefuroxime and third-generation cephalosporins (ceftriaxone, ceftazidime and cefotaxime) (Laving et al., 2003). The variation in resistance patterns is likely to reflect temporal and geographic differences; hence the need for local surveillance data to support empiric antibiotic prescriptions.

### 3.5 Identification of severe bacterial illness

#### 3.5.1 Clinical predictors

A total of eight studies from India (Bang et al., 2005, Gupta et al., 2000), Papua New Guinea (Duke et al., 2005, Weber et al., 2003), Kenya (English et al., 2003, English et al., 2004), The Gambia/The Philippines/Ethiopia (Weber et al., 2003), Australia (Hewson et al., 2000) and Bangladesh (Kalter et al., 1997) were reviewed to determine which clinical signs best predict severe bacterial illness (SBI) in young infants. Three of the studies (Gupta et al., 2000, Hewson et al., 2000, Kalter et al., 1997) evaluated the validity of previously identified markers of serious illness in infancy. Overall, the following panel of signs are likely to be the most valuable in identifying a young infant at risk of severe illness: cyanosis (Duke et al., 2005, English et al., 2004, Weber et al., 2003); a history of feeding difficulty (Bang et al., 2005, Duke et al., 2005, English et al., 2003, English et al., 2004, Weber et al., 2003); breathing difficulty (grunting) (English et al., 2004, Hewson et al., 2000, Weber et al., 2003); fast breathing (respiratory rate >60 bpm) (Bang et al., 2005, Duke et al., 2005, English et al., 2004, Weber et al., 2003); abnormal behaviour (English et al., 2003, English et al., 2004, Weber et al., 2003); and fever/temperature >38 °C (Hewson et al., 2000, Weber et al., 2003).

#### 3.5.2 Baby Check

Three studies investigated the utility of the Baby Check, a systematic way of grading the severity of illness in infants younger than 6 months (Chandran et al., 1998, Morley et al., 1994, Twite and Morley, 1995). The scoring system involves the recognition of a combination of seven symptoms and 12 signs, with the higher the total score the greater the chance of the baby being seriously ill. In the reviewed studies, the scores generally correlated with the grades of illness, suggesting that the use of the Baby Check increases the accuracy of identifying sick infants. Furthermore, these findings seem to be stable over different settings (Twite and Morley, 1995). However, a number of factors limit the utility of the Baby Check as a tool to grade the severity of the illness: (1) there was no laboratory- or investigation-confirmed ‘gold standard’ against which to test the accuracy of the Baby Check; (2) the scoring system seems to be inadequate in cases of co-morbidity (many young sick infants present with multiple illnesses concurrently); and (3) the Baby Check demands a long list of values and a numerical scoring system, making it a bit impractical for primary health care settings in LMICs. Despite these limitations, the scoring system seems to be fairly accurate in identifying high-risk infants.

## 4 Discussion

### 4.1 Summary of current evidence

The study setting has important implications in young infant infections: most hospital-based studies even in LMICs are conducted in neonatal intensive care units (NICUs), with higher proportions of pre-term births, prolonged hospital stays and high antibiotic resistance rates. However, adequate laboratory testing is not feasible in the community, with consequences of disease misclassification in most community-based studies. These issues notwithstanding, reviewed evidence shows that causative agents of young infant sepsis vary according to the setting within which infection is acquired: gram-negative organisms (*E. coli*, *Klebsiella*, *Pseudomonas*) predominate in hospital-acquired infections, while gram-positive organisms (*Staph. aureus*, GBS, *Strep. pneumoniae*, *Strep. pyogenes*) predominate in community-acquired infections. Not surprisingly, common organisms in mixed-site infections are variable, with *E. coli*, *Klebsiella*, *Staph. aureus* and GBS being important organisms. On the whole, *E. coli*, *Klebsiella*, *Pseudomonas*, *Staph. aureus* and *GBS* are responsible for most infections across all the settings. Causative organisms in EOS and LOS are variable, reflecting differences in definitions, in population characteristics and in predisposing factors: GBS is commonly reported in EOS (signifying vertical transmission from the maternal genital tract), whereas *E. coli* and *Pseudomonas* are frequent in both EOS and LOS. These findings underscore the need to tailor empiric antibiotic therapy according to the site at which the infection is acquired.

Based on the available evidence, the combination of ampicillin or penicillin and gentamicin still remains suitable for first-line treatment for young infant sepsis. However, the rapid emergence of resistance to third-generation cephalosporins (Musoke and Revathi, 2000) and recent findings from the USA that suggest that first-line treatment with these drugs is associated with an increased risk of mortality (Clark et al., 2006) caution against their overuse and provide a strong rationale for improving local surveillance on antibiotic susceptibility and outcomes to guide future empiric therapy.

Evidence from studies that examined clinical recognition of severe illnesses suggest that any one of cyanosis, a history of feeding difficulty, breathing difficulty (grunting), fast breathing (respiratory rate >60 bpm), chest indrawing, abnormal behaviour/change in activity and fever/temperature >38 °C seem to be sensitive, although their specificity is modest. Unfortunately, while none of these signs alone reliably predicts SBIs, demanding that a young infant have two or more signs considerably reduces sensitivity, although it does improve the positive predictive value. In conclusion, currently, there seems to be no highly sensitive and highly specific clinical screening tool for identification of seriously sick infants with sepsis. At present, therefore, optimizing sensitivity and simplicity of classification to avoid potentially life-threatening disease would suggest categorizing a young infant as at high risk of SBI based on any one of the signs listed above.

### 4.2 Conclusions

In most of the reported studies, it is difficult to determine whether infections were ‘maternally-acquired’ (a consequence of exposure before birth or within the birth canal), ‘hospital-acquired’ (a consequence of any of poor delivery techniques, poor ward hygiene or prolonged stay) or ‘community-acquired’ (a consequence of exposure in an entirely community setting). Also, there were wide variations in the definitions for EOS/LOS, clinical signs and age distribution of ‘young’ infants: 0–28 d (Aurangzeb and Hameed, 2003, Bang et al., 2005, Laving et al., 2003, Mokuolu et al., 2002, Waheed et al., 2003); 0–60 d (Adejuyigbe et al., 2004, Berkley et al., 2005, Duke et al., 2005, Milledge et al., 2005, English et al., 2004, Gupta et al., 2000, Kalter et al., 1997, Weber et al., 2003); 0–90 d (English et al., 2003, WHO Young Infants Study Group, 1999); 1–26 weeks (Hewson et al., 2000). Future work would benefit considerably from a clear description of the study setting and probable source of infection and increasing use of enhanced antibiotic susceptibility testing. Moreover, the observed variations in the definitions highlight the necessity for a consensus definition of paediatric sepsis, to aid in standardization of observational studies and evaluation of age-specific therapeutic hypotheses in clinical trials.

A number of important knowledge gaps emerged in this review: first, there is a need for routine longitudinal surveillance across settings to describe the varied pathogens causing young infant sepsis, as well as their changing susceptibility profile. Such surveillance should then direct future randomized controlled trials comparing clinical failure rates and antibiotic regimens for suspected sepsis in young infants. Secondly, more rigorous field-tested studies are needed to evaluate various signs as predictors of SBIs in young infants. These studies should give rise to simple, robust clinical prediction rules that can alert mothers and caretakers to seek care and be used in essential training packages for primary health care workers.

## Authors’ contributions

ME conceived the idea for the review; ON conducted the literature searches; ON and ME reviewed articles and assessed their quality, and drafted and finalized the manuscript. ON and ME are guarantors of the paper.

## Funding

This work was supported under a Wellcome Trust Senior Fellowship awarded to Mike English (#076827).

## Conflicts of interest

None declared.

## Ethical approval

Not required.
